# Improved Outcome Prediction Using CT Angiography in Addition to Standard Ischemic Stroke Assessment: Results from the STOPStroke Study

**DOI:** 10.1371/journal.pone.0030352

**Published:** 2012-01-20

**Authors:** R. Gilberto González, Michael H. Lev, Gregory V. Goldmacher, Wade S. Smith, Seyedmehdi Payabvash, Gordon J. Harris, Elkan F. Halpern, Walter J. Koroshetz, Erica C. S. Camargo, William P. Dillon, Karen L. Furie

**Affiliations:** 1 Department of Radiology, Massachusetts General Hospital and Harvard Medical School, Boston, Massachusetts, United States of America; 2 Department of Neurology, University of California San Francisco, San Francisco, California, United States of America; 3 Department of Radiology and Institute for Technology Assessment, Massachusetts General Hospital and Harvard Medical School, Boston, Massachusetts, United States of America; 4 National Institute of Neurological Disorders and Stroke, Bethesda, Maryland, United States of America; 5 Department of Neurology, Massachusetts General Hospital and Harvard Medical School, Boston, Massachusetts, United States of America; 6 Department of Radiology, University of California San Francisco, San Francisco, California, United States of America; Innsbruck Medical University, Austria

## Abstract

**Purpose:**

To improve ischemic stroke outcome prediction using imaging information from a prospective cohort who received admission CT angiography (CTA).

**Methods:**

In a prospectively designed study, 649 stroke patients diagnosed with acute ischemic stroke had admission NIH stroke scale scores, noncontrast CT (NCCT), CTA, and 6-month outcome assessed using the modified Rankin scale (mRS) scores. Poor outcome was defined as mRS>2. Strokes were classified as “major” by the (1) Alberta Stroke Program Early CT Score (ASPECTS+) if NCCT ASPECTS was≤7; (2) Boston Acute Stroke Imaging Scale (BASIS+) if they were ASPECTS+ or CTA showed occlusion of the distal internal carotid, proximal middle cerebral, or basilar arteries; and (3) NIHSS for scores>10.

**Results:**

Of 649 patients, 253 (39.0%) had poor outcomes. NIHSS, BASIS, and age, but not ASPECTS, were independent predictors of outcome. BASIS and NIHSS had similar sensitivities, both superior to ASPECTS (p<0.0001). Combining NIHSS with BASIS was highly predictive: 77.6% (114/147) classified as NIHSS>10/BASIS+ had poor outcomes, versus 21.5% (77/358) with NIHSS≤10/BASIS− (p<0.0001), regardless of treatment. The odds ratios for poor outcome is 12.6 (95% CI: 7.9 to 20.0) in patients who are NIHSS>10/BASIS+ compared to patients who are NIHSS≤10/BASIS−; the odds ratio is 5.4 (95% CI: 3.5 to 8.5) when compared to patients who are only NIHSS>10 or BASIS+.

**Conclusions:**

BASIS and NIHSS are independent outcome predictors. Their combination is stronger than either instrument alone in predicting outcomes. The findings suggest that CTA is a significant clinical tool in routine acute stroke assessment.

## Introduction

The Screening Technology and Outcomes Project in Stroke (STOPStroke) was prospective study whose purpose was to evaluate advanced CT technology in ischemic stroke. Computed tomography angiography (CTA) is a highly reliable method to detect occlusions of the major cerebral arteries that are occluded in major ischemic stroke syndromes. [1] Recent evidence suggests that it may also be useful in identifying patients for thrombolytic therapy. [2] Yet despite its wide availability, CTA has yet to be incorporated in the routine evaluation of ischemic stroke patients or in stroke classification. Classification instruments are valuable in ischemic stroke for prognosis, assessing current practices, and evaluation of novel therapies. Clinical instruments such as the National Institutes of Health Stroke Scale (NIHSS) have proven value in predicting stroke outcomes and assessing new treatments. [3] However, they do not provide direct information on the initial event (arterial occlusion) that produces the stroke syndrome and cerebral infarction. This limits their usefulness in assessing the efficacy of therapies targeting vascular occlusion. [4]

The most widely employed imaging-based classification instrument, the Alberta Stroke Program Early CT Score (ASPECTS) system, is based on non-contrast CT (NCCT) [5] scoring of parenchymal changes, but does not evaluate arterial occlusion. The Boston Acute Stroke Imaging Scale (BASIS) combines vascular and parenchymal imaging. [6] The analysis presented here compares BASIS, ASPECTS and NIHSS classification instruments in prospectively enrolled patients in the STOPStroke study. All patients in the STOPstroke cohort underwent NCCT and CTA at the time of initial evaluation for ischemic stroke. In addition, we assessed whether stroke classification that involved combining neurological and CTA information could improve stroke outcome prediction.

## Methods

The prospective STOPStroke study received institutional review board approval at both institutions, and was Health Insurance Portability and Accountability Act compliant. All patients gave informed written consent.

### Patient cohort

742 consecutive patients presenting to the Massachusetts General Hospital and the University of California at San Francisco were prospectively enrolled in the Screening Technology and Outcomes Project in Stroke (STOPStroke). Patients suspected of having ischemic stroke within 24 hours of symptom onset underwent emergency NCCT followed immediately by CTA. Patients were excluded if iodinated contrast agent was contraindicated or if there was intracranial hemorrhage. Demographic data, past medical history, and NIHSS scores were obtained at admission. Modified Rankin scale (mRS) scores were obtained at 6 months. [7], [8], [9] Favorable outcome was defined as mRS = 0–2, and poor outcome as mRS>2. Patients were excluded if reliable mRS or NIHSS scores were not obtained.

### Scanning procedures

NCCT and CTA were performed according to standard protocols with multidetector CT scanners (LightSpeed; GE Healthcare, Chalfont St. Giles, UK). [1] Representative NCCT parameters were as follows: 120–140 kVp, 170 mA, 2-second scan time, and 5-mm section thickness. Biphasic helical CTA scanning, at the same head tilt, was performed immediately afterward, with 100–140 ml of contrast (Isovue; Bracco Diagnostics, Princeton, NJ) at 3 ml/sec and a 25-second delay (40 seconds for patients in atrial fibrillation). Contrast allergy and renal dysfunction are the major relative contra-indications to contrast administration. Parameters were 140 kVp, 220–250 mA, 0.8–1.0-second rotation, 2.5-mm section thickness, 1.25-mm reconstruction interval, 3.75-mm/rotation table speed, and 0.75∶1 pitch. Source images were reconstructed into standardized maximum intensity projections of the intracranial and extracranial vasculature.

CTA information results were available to the attending stroke physicians. However, information is not available on how it may have influenced treatment strategies.

### Image Review

Image review was independently performed on a workstation (Impax; Agfa Technical Imaging Systems, Richfield Park, NJ) by neuroradiologists or neurologists (M.H.L., E.C., and W.J.K.) as previously described. [10] Reviewers had information on patient age, sex, and presenting clinical symptoms but were blinded to all information after the initial emergency evaluation. [11] NCCT images were reviewed first, followed by CTA. Disagreements were resolved by consensus. The reviewers recorded both arterial occlusions and brain areas with hypodensity considered to have resulted from acute ischemia.

### Stroke Severity Classification

**Figure 1:**
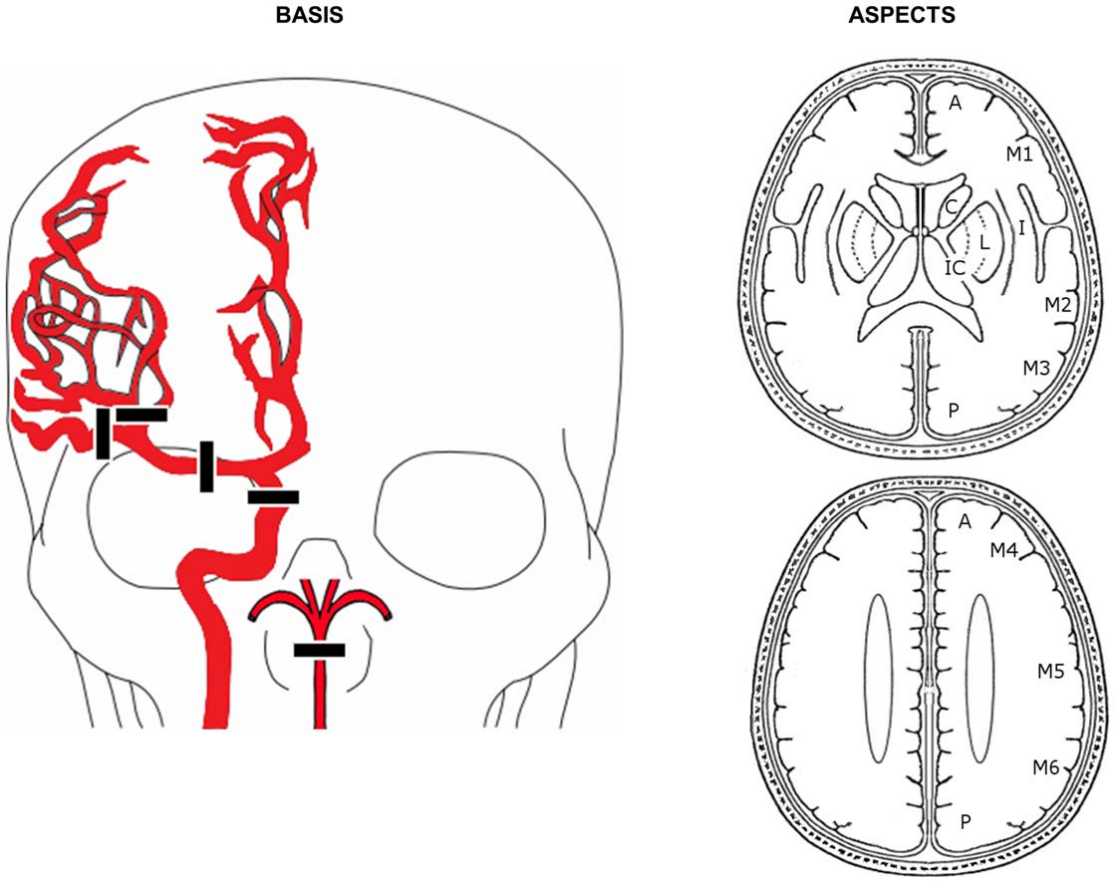
BASIS and ASPECTS classification. Patients are classified as BASIS+ if there are proximal cerebral artery occlusions observed on CTA or a significant hypodensities on NCCT. The relevant arterial segment occlusions are depicted in drawing on the left and are defined as including the following arteries: distal (intracranial) internal carotid artery (ICA), proximal (M1 or M2) middle cerebral artery (MCA) and/or basilar artery (BA). If none of these arteries are observed to be occluded on the CTA, then the NCCT is scored using the scheme shown on the right for anterior circulation strokes, which is also used for ASPECTS scoring. If a hypodensity deemed to be consistent with acute ischemic infarction is identified in one of the cerebral regions shown, a point is deducted from the maximum score of 10. Patients with scores of 7 or less are both BASIS+ and ASPECTS+. BASIS+ classification for posterior circulation strokes in the absence of basilar artery occlusion requires bilateral pons or bilateral thalamus hypodensities.

The BASIS classification system was devised after all patients were enrolled in STOPStroke, and was validated in 2 independent retrospective studies. [6], [12] Figure 1 illustrates patient classification by the neuroimaging instruments evaluated here. Strokes were classified as major by BASIS (BASIS+) if occlusion was identified in the distal ICA, proximal MCA (M1 or M2 segments), or the basilar artery, or if early ischemic changes were identified in 3 or more ASPECTS territories in the anterior circulation, or as previously described for posterior circulation ischemia. [6] Strokes were classified as major by ASPECTS (ASPECTS+) if early ischemic changes were identified in 3 or more ASPECTS territories (ASPECTS score ≤7). [13] Based in part on methodology used by Ingall et al, [14] a dichotomized NIHSS was employed with major stroke classification given to patients with an admission NIHSS of greater than 10 (designated NIHSS>10).

### Statistical analysis

Sensitivity, specificity, positive predictive value (PPV), negative predictive value (NPV) and accuracy for prediction of poor outcome were determined for BASIS, ASPECTS, and dichotomized NIHSS classifications. Comparisons between the sensitivities and specificities of classification systems for predicting poor outcome were conducted using McNemar's Test. [15] Other comparisons were tested for statistical significance using 3×2 or 2×2 contingency tables, the t-test, odds ratios, or Wilcoxon's Rank Sum test, as appropriate. BASIS, NIHSS, ASPECTS, and patient age were evaluated as predictors of poor outcome using forced entry and stepwise multivariate logistic regression models (STATA 10, StataCorp LP).

## Results

### Cohort Characteristics

A total of 742 patients were enrolled. Ninety were excluded for lack of a reliable mRS at 6 months and 3 were excluded for lack of reliable NIHSS scores at admission. 649 patients formed the analyzed cohort. Table 1 shows patient demographics, comorbidities, and treatment. Significant differences between patients classified as major strokes by the 3 classification instruments and the remainder of the cohort included a higher prevalence of atrial fibrillation, a higher median NIHSS, higher use of thrombolytic therapy, and a much higher number of poor outcomes. Males were less likely to have major strokes classified by all 3 instruments, but this was statistically significant only for BASIS and ASPECTS classification. Hypertension was significantly less prevalent in strokes classified as major by ASPECTS only.

**Table 1:** Demographics, comorbidities, and treatment.

	All patients	NIHSS>10	NIHSS≤10	p-value	BASIS+	BASIS−	p-value	ASPECTS+	ASPECTS-	p-value
n	649	188	461	n/a	249	400	n/a	121	528	n/a
Age (mean±SD)	68.2±15.4	69.5±16.6	67.8±15.0	0.11	68.2±16.8	68.2±14.6	0.892	65.9±18.3	68.8±14.7	0.112
Male sex	330(50.8%)	88(46.8%)	242(52.5%)	0.189	108(43.4%)	222(55.5%)	**0.001**	50(41.3%)	280(53.0%)	**0.02**
NIHSS (median)	5	16	3	n/a	12	3	**0.000**	14	4	**0.000**
Diabetes	120(18.5%)	38(20.2%)	82(17.8%)	0.47	44(17.7%)	76(19.0%)	0.671	23(19.0%)	97(18.4%)	0.871
CAD	147(22.7%)	47(25.0%)	100(21.7%)	0.361	58(23.3%)	89(22.3%)	0.758	26(21.5%)	121(22.9%)	0.735
Atrial fibrillation	137 (21.1%)	59(31.4%)	78(16.9%)	**0.000**	75(30.1%)	62(15.5%)	**0.000**	36(29.8%)	101(19.1%)	**0.01**
Smoking	201(31.0%)	51(27.1%)	150(32.5%)	0.176	74(29.7%)	127(31.8%)	0.586	36(29.8%)	165(31.3%)	0.748
Hyperlipid	190(29.3%)	52(27.7%)	138(29.9%)	0.563	76(30.5%)	114(28.5%)	0.582	36(29.8%)	154(29.2%)	0.898
IV tPA	101(15.6%)	64(34.0%)	37(8.0%)	**0.000**	69(27.7%)	32(8.0%)	**0.000**	34(28.1%)	67(12.7%)	**0.000**
IA thrombolysis	31(4.8%)	29(15.4%)	2(0.4%)	**0.000**	31(12.4%)	0(0.0%)	**0.000**	12(9.9%)	19(3.6%)	**0.003**

61% (396/649) of the patients had favorable outcomes and 39% (253/649) had poor outcomes. Of the poor outcomes, 54.5% (138/253) had NIHSS>10 and 59.7% (151/253) were BASIS+, an insignificant difference (p = 0.57). However, only 30.8% (78/253) of patients that had poor outcomes were ASPECTS+. This number was significantly lower than the other 2 classification instruments (both p<0.0001).

BASIS+ patients had major artery occlusion and/or parenchymal ischemic changes. Of the 253 BASIS+ patients, 200 had arterial occlusions. Poor outcomes were found in 126 patients (63%). While the proportion of patients with poor outcomes was slightly higher than BASIS+ patients, there were 25 additional patients that had poor outcomes identified by the BASIS instrument.

### Predictive Power of Classification Instruments

Table 2 displays the sensitivity, specificity, PPV, NPV, and accuracy for prediction of poor outcomes by BASIS, ASPECTS, and admission NIHSS>10. Classification by both BASIS and dichotomized NIHSS is significantly more sensitive than ASPECTS (p<0.0001). ASPECTS is significantly more specific than BASIS (p<0.0001), but not more than dichotomized NIHSS. A trend was found with BASIS slightly more sensitive than dichotomized NIHSS (p = 0.06). NIHSS>10 was significantly more specific than BASIS (p = 0.03). In univariate analysis, BASIS (p = 0.0217) and NIHSS (p<0.001) were both significant predictors of poor outcome In the multivariate forced entry and stepwise logistic models, BASIS, NIHSS, and patients' age were independent predictors of poor outcome (all p<0.004), however ASPECTS was not (p = 0.18). The odds ratios for ASPECTS, BASIS, NIHSS, and age were 1.5±0.5, 2.1±0.5, 6.3±1.5, and 1.1±0.01, respectively. There were no significant interactions between each of these variables.

**Table 2:** Prediction of poor outcome.

	NIHSS>10	BASIS	ASPECTS
Sensitivity	54.5	59.7	30.0
Specificity	87.4	75.3	88.6
PPV	73.4	60.6	62.8
NPV	75.1	74.5	66.5
Accuracy	74.6	69.2	65.8

### Combined NIHSS and BASIS Classification Instrument

**Figure 2:**
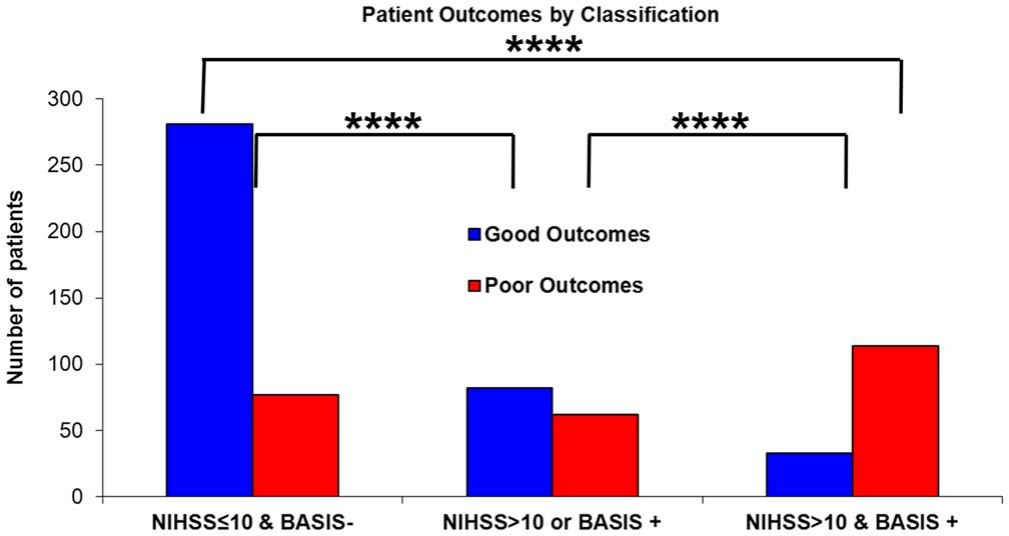
Patient outcomes by NIHSS/BASIS classification. Patient outcomes, regardless of treatment, are grouped into possible combinations of BASIS and NIHSS. There are significant differences in outcomes amongst the categories (3×2 contingency table p<0.0001). Both the NIHSS≤10/BASIS− and the NIHSS>10/BASIS+ groups are significantly different from each other and from the other categories (****, p<0.0001).

Because BASIS and NIHSS were shown by logistic regression to be independently significant predictors of poor outcome, a classification scheme combining BASIS and NIHSS was evaluated. 77.8% (505/649) of all patients were classified as NIHSS≤10 and BASIS− or NIHSS>10 and BASIS+. The predictive power of this combined instrument is displayed in Figure 2. Of patients classified as NIHSS≤10 and BASIS−, 78.5% (281/358) had good outcomes regardless of treatment, which is a significantly higher proportion than patients in the other groups (3×2 contingency p<0.0001; vs. each class p<0.0001). Conversely, 77.6% (114/147) with NIHSS>10 and BASIS+ had poor outcomes (vs. each class p<0.0001) also without regard to treatment. The odds ratios for poor outcome is 12.6 (95% CI: 7.9 to 20.0) in patients who are NIHSS>10/BASIS+ compared to patients who are NIHSS≤10/BASIS−; the odds ratio is 5.4 (95% CI: 3.5 to 8.5) when compared to patients who are only NIHSS>10 or BASIS+.

### IV tPA, Endovascular Therapy, Classification and Outcomes

The effect of thrombolytic therapy on outcome prediction by the 3 classification instruments was evaluated. 101 patients (15.6%) received IV tPA (86 received IV tPA only, 15 received both IV tPA and endovascular therapy). 51 of these patients had poor outcomes. The proportion of poor outcomes in patients who received IV tPA was significantly higher than the proportion of poor outcomes in patients who did not receive tPA (50.5% vs. 32.8% (180/548); p<0.001). However, 342 of the untreated patients had mild clinical symptoms (NIHSS≤5). If this subset is removed, a slightly higher proportion of patients who received IV tPA had good outcomes (43/91; 47.3%) than those who did not receive this treatment (92/206; 44.7%), but the difference was not significant (p = 0.68). The majority of the 101 patients who received IV tPA were BASIS+ (69/101) or NIHSS>10 (64/101), while only about one-third were ASPECTS+ (34/101). Despite IV tPA treatment, most BASIS+ (39/69, 56.5%), NIHSS>10 (41/64, 64%), and ASPECTS+ (21/34, 62%) patients had poor outcomes.

A total of 31 patients (4.8%) underwent endovascular therapy including 15 who received IV tPA prior to the procedure. Patients in this group had the worst outcomes with 23 (74.2%) having mRS>2 at 6 months. These patients also had the most severe symptoms with a median NIHSS of 18, compared to a median NIHSS of 5 for the remainder of the cohort (p<0.001). With respect to the classification instruments, 31/31 were BASIS+, 14/31 were ASPECTS+ and 29/31 were NIHSS>10.

## Discussion

Contemporary CT can rapidly produce images that reliably depict major cerebral artery occlusion. The STOPStroke study demonstrates that CTA acquisition does not hinder stroke patient management, and that additional information provided by CTA may enhance patient care. Combining the imaging-based BASIS classification instrument with the NIHSS substantially enhances outcome prediction, and may help in identifying subsets of patients that are more or less likely to benefit from therapy.

Among vessel imaging methods, CTA is more reliable than MRA because it is less susceptible to motion, pulsation, flow, and other artifacts, and more reliable than ultrasound because the latter is often limited in coverage due to calcification/tortuous or deep vessels/overlying bone. There were no relevant side effects of the iodinated intravenous CT contrast in this patient cohort, as reported in a publication obtained in the same patient cohort. [16]

That there is a clinical need for refinement of the existing classification instruments used in the evaluation and therapeutic management of acute stroke patients - most notably the NIH stroke scale and ASPECT scores - is supported by the current DIAS III and IV study designs, which use CTA as a critical component of patient selection. [17]NIHSS has been shown to predict length of stay, hospital cost, clinical outcomes, and hospital discharge disposition. [3], [18], [19], [20] However, the NIHSS and similar instruments do not identify the occluded artery, the initiating event that leads to neurological symptoms. As noted by Caplan, the lack of information on arterial occlusion has likely limited progress in the treatment of ischemic stroke. [4] Neuroimaging overcomes this limitation. Several stroke classification systems that employ imaging have been described. [5], [21], [22], [23] However, BASIS [6] is the only instrument that incorporates angiographic data, is independent of whether CT or MRI is used, and classifies patients with either anterior or posterior circulation strokes. The use of CTA has previously been shown to predict outcomes, [24], [25], [26] and BASIS was built upon that foundation.

The majority of patients in this study had mild neurological symptoms (54.6% had NIHSS 0–5), and good outcomes (61%). Patients classified with severe strokes by all 3 instruments were found to have a higher prevalence of atrial fibrillation, higher use of thrombolytic therapy, and a much higher proportion of poor outcomes. BASIS and NIHSS were more sensitive than ASPECTS for prediction of poor outcomes, probably due to the poor sensitivity of NCCT for identifying early ischemia. While BASIS+ and NIHSS>10 have similar power in predicting poor outcomes, they are not equivalent, and they are independent predictors of poor outcomes. Some have suggested that NIHSS>10 is predictive major artery occlusion. This was not found to be the case in the present study in which 185 patients had NIHSS>10, but only122 of these had a major artery occlusion.

Combined NIHSS/BASIS classification is substantially more powerful in predicting outcomes than any single classification instrument. Close to 80% of all patients were dual-classified as either NIHSS≤10/BASIS− or NIHSS>10/BASIS+, with nearly 80% of NIHSS≤10/BASIS− patients having a good outcomes and a similar percentage of NIHSS>10/BASIS+ patients having poor outcomes, regardless of treatment. The potential prognostic clinical utility of this classification is substantial as indicated by the odds ratio of a poor outcome of over 12 in NIHSS>10/BASIS+ patients when compared to those classified as NIHSS≤10/BASIS−.

The predictive efficacy of combining NIHSS and BASIS into a single classification instrument is reasonable given our current understanding of ischemic stroke. BASIS is a measure of the early physiological abnormalities underlying the ischemic stroke process while the NIHSS reflects the functional significance of the same process. It is the amalgamation of this complementary information that makes the combined classification scheme potent in predicting the functional status of the patient several months after the event. For example, a NIHSS>10/BASIS+ classification indicates that the pathophysiology of a major stroke is present (either occlusion of a major cerebral artery, substantial parenchymal injury or both), and that this abnormal physiology is functionally severe. In the absence of therapy, the expected outcome would be a major cerebral infarction producing a severe functional disability.

The improvement of predictive power in ischemic stroke through the combination of imaging information with clinical assessments to has been previously demonstrated in transient ischemic attacks by 2 groups. [27], [28] Both groups have shown that the presence of a DWI abnormality greatly improves the prediction of the early risk of stroke after TIA. The similarity to the work presented here is that imaging provides physiological information that is complementary to the clinical evaluation.

The STOPstroke study has demonstrated that CT angiography can be performed while maintaining a high percentage of patients that receive thrombolytic therapy, and the imaging data can be used for classification that can predict outcomes independent of neurological evaluation. Classifications by NIHSS and BASIS are superior to ASPECTS, and the combination of NIHSS and BASIS instruments is substantially more powerful than any single instrument. These observations may be explained by the early identification by neuroimaging of stroke pathophysiology that produces functionally severe symptoms, a hypothesis that merits further investigation. The use of combined NIHSS/BASIS classification may permit the design of efficient prospective trials of stroke treatment.
